# Zika a Vector Borne Disease Detected in Newer States of India Amidst the COVID-19 Pandemic

**DOI:** 10.3389/fmicb.2022.888195

**Published:** 2022-06-10

**Authors:** Pragya D. Yadav, Harmanmeet Kaur, Nivedita Gupta, Rima R. Sahay, Gajanan N. Sapkal, Anita M. Shete, Gururaj R. Deshpande, Sreelekshmy Mohandas, Triparna Majumdar, Savita Patil, Priyanka Pandit, Abhinendra Kumar, Dimpal A. Nyayanit, K. H. Sreelatha, S. Manjusree, Hiba Sami, Haris Mazoor Khan, Anuradha Malhotra, Kanwardeep Dhingra, Ravisekhar Gadepalli, V. Sudha Rani, Manoj Kumar Singh, Yash Joshi, Manisha Dudhmal, Nandini Duggal, Mala Chabbra, Lalit Dar, Pranita Gawande, Jyoti Yemul, Kaumudi Kalele, Rajalakshmi Arjun, K. Nagamani, Biswa Borkakoty, Ganesh Sahoo, Ira Praharaj, Shanta Dutta, Pradip Barde, S. C. Jaryal, Vinita Rawat

**Affiliations:** ^1^Indian Council of Medical Research, National Institute of Virology, Pune, India; ^2^Indian Council of Medical Research, V. Ramalingaswami Bhawan, New Delhi, India; ^3^Virus Research and Diagnostic Laboratory, Government Medical College, Thiruvananthapuram, India; ^4^Virus Research and Diagnostic Laboratory, Jawaharlal Nehru Medical College, Aligarh, India; ^5^Virus Research and Diagnostic Laboratory, Government Medical College, Amritsar, India; ^6^Virus Research and Diagnostic Laboratory, All India Institute of Medical Sciences, Jodhpur, India; ^7^Virus Research and Diagnostic Laboratory, Osmania Medical College Hyderabad, Hyderabad, India; ^8^Virus Research and Diagnostic Laboratory, Rajendra Institute of Medical Sciences, Ranchi, India; ^9^Virus Research and Diagnostic Laboratory, Atal Bihari Vajpayee Institute of Medical Sciences & Dr. Ram Manohar Lohia Hospital, New Delhi, India; ^10^Virus Research and Diagnostic Laboratory, All India Institute of Medical Sciences, New Delhi, India; ^11^Kerala Institute of Medical Sciences, Thiruvananthapuram, India; ^12^Virus Research and Diagnostic Laboratory, Gandhi Medical College, Secunderabad, India; ^13^Virus Research and Diagnostic Laboratory, ICMR-Regional Medical Research Centre, Dibrugarh, India; ^14^Virus Research and Diagnostic Laboratory, ICMR-Rajendra Memorial Research Institute of Medical Sciences, Patna, India; ^15^Virus Research and Diagnostic Laboratory, ICMR-Regional Medical Research Centre, Bhubaneswar, India; ^16^Virus Research and Diagnostic Laboratory, ICMR-National Institute of Cholera and Enteric Diseases, Kolkata, India; ^17^Virus Research and Diagnostic Laboratory, ICMR-National Institute of Research in Tribal Health, Jabalpur, India; ^18^Virus Research and Diagnostic Laboratory, Dr. Rajendra Prasad Government Medical College, Tanda, India; ^19^Virus Research and Diagnostic Laboratory, Government Medical College, Haldwani, India

**Keywords:** Zika virus, India, microcephaly, next generation sequencing, dengue serotype

## Abstract

**Background:**

During the second wave of the COVID-19 pandemic, outbreaks of Zika were reported from Kerala, Uttar Pradesh, and Maharashtra, India in 2021. The Dengue and Chikungunya negative samples were retrospectively screened to determine the presence of the Zika virus from different geographical regions of India.

**Methods:**

During May to October 2021, the clinical samples of 1475 patients, across 13 states and a union territory of India were screened and re-tested for Dengue, Chikungunya and Zika by CDC Trioplex Real time RT-PCR. The Zika rRTPCR positive samples were further screened with anti-Zika IgM and Plaque Reduction Neutralization Test. Next generation sequencing was used for further molecular characterization.

**Results:**

The positivity was observed for Zika (67), Dengue (121), and Chikungunya (10) amongst screened cases. The co-infections of Dengue/Chikungunya, Dengue/Zika, and Dengue/Chikungunya/Zika were also observed. All Zika cases were symptomatic with fever (84%) and rash (78%) as major presenting symptoms. Of them, four patients had respiratory distress, one presented with seizures, and one with suspected microcephaly at birth. The Asian Lineage of Zika and all four serotypes of Dengue were found in circulation.

**Conclusion:**

Our study indicates the spread of the Zika virus to several states of India and an urgent need to strengthen its surveillance.

## Introduction

Zika virus (ZIKV), a vector-borne flavivirus transmitted by the bite of infected *Aedes* mosquitoes, mainly *Aedes aegypti* and *Aedes albopictus*. ZIKV was first isolated from the Zika forest of Africa in 1947 from the serum of rhesus monkey during yellow fever (YF) surveillance (Dick, 1952). The spectrum of the Zika virus disease (ZVD) is similar to diseases caused by other flaviviruses, namely, Dengue virus (DENV), West Nile virus (WNV), Yellow Fever (YF), and Japanese encephalitis (JE) with an incubation period of 3–14 days (Pielnaa et al., 2020). The virus is also known to spread through sexual, vertical, and blood transfusion (Rothan et al., 2018). The first human case of ZIKV was reported in 1952 and thereafter, for several decades, sporadic cases of mild illness were reported from Africa and Asia. First major outbreaks were reported from Yap (Micronesia) in 2007 and French Polynesia (2013–2014) (Moore et al., 1975; Duffy et al., 2009; Musso et al., 2018). The clinical spectrum of ZVD usually consists of mild illness including fever, myalgia, headache, rash, arthritis, and conjunctival congestion with up to 4/5th of the infected individuals being asymptomatic (Duffy et al., 2009; Calvet et al., 2016). In 2016, a large-scale outbreak of ZVD in Brazil was reported, which was found to be associated with microcephaly and birth defects in newborn babies (Rocha et al., 2019). This was the first report of ZIKV, a relatively innocuous virus, causing severe outcomes. ZVD was also implicated to cause Guillain Barre Syndrome (GBS) (Cao-Lormeau et al., 2016). Therefore, the World Health Organization declared ZVD as a public health emergency of international concern in February 2016 (Zanluca et al., 2015; Heukelbach et al., 2016).

India initiated sentinel surveillance of ZVD in March 2016 through its network of virus research and diagnostic laboratories (VRDLs) by the Indian Council of Medical Research (ICMR) across the country (Department of Health Research, 2022). The sentinel ZIKV surveillance was initiated with 10 laboratories in 2016, the VRDLs were up-scaled to 56 in 2018 and 132 by 2021. The trained VRDLs were advised to test at least 10 dengue virus (DENV) and chikungunya virus (CHIKV) negative samples for ZIKV, throughout the year. Molecular testing reagents for ZIKV were provided to all VRDLs by the ICMR-National Institute of Virology (NIV), Pune. The laboratory-based surveillance also focused on the newborn birth defect screening in 55 tertiary hospitals in India (Gupta et al., 2020). Through this surveillance, sporadic cases were first picked up in the state of Gujarat (2016−2017) and Tamil Nadu (2017). Following this, outbreaks of ZIKV were detected in Rajasthan and Madhya Pradesh in 2018 (Sapkal et al., 2018; Yadav et al., 2019; Malhotra et al., 2020). After 2020, the public health surveillance of ZIKV could not be continued with the same vigor due to the involvement of all VRDLs in COVID-19 diagnostics considering the subsequent waves of the pandemic. All these VRDLs were advised to store the DENV/CHIKV negative samples for ZIKV testing in the future.

However, in 2021, ZIKV outbreaks were reported in Kerala (May–July), Maharashtra (July), and Uttar Pradesh (October) states of India (Yadav et al., 2022). Since these outbreaks were reported from distant locations and over a period of 6 months, we conducted a retrospective screening of dengue and chikungunya negative clinical samples (stored with VRDLs), from May to October 2021, to understand the extent of the spread of ZIKV in India. In this article, we have described the results and conclusions of this retrospective analysis, which revealed the circulation of ZIKV in Delhi, Jharkhand, Rajasthan, Punjab, and Telangana states of India in 2021 in addition to Kerala, Maharashtra, and Uttar Pradesh. Co-infection of Zika and dengue and chikungunya were another concern in many places.

## Materials and Methods

### Ethics Statement

The study was approved by the Institutional Human Ethics Committee at the (IHEC Number-NIV/IEC/Mar/2021/D-9 dated 9th April 2021) and the Institutional Biosafety Committee at ICMR-NIV, Pune.

### Sample Type

The retrospective serum, urine, plasma, and whole blood samples from May to October 2021 were tested for ZIKV as per the following inclusion criteria: (a) collected in the acute phase of infection (within 7 days of onset of illness); (b) screened for DENV and CHIKV by IgM ELISA; and (c) stored at –20°C at the respective centers.

### Sites Under the Study

A total of 1520 clinical samples, serum (1253), plasma (99), whole blood (120), and urine (48) were collected from 1,475 patients across 16 VRDLs from 14 States/Union Territories (UTs) in India [Delhi, Kerala, Punjab, Rajasthan, Uttar Pradesh (UP), Uttarakhand, Madhya Pradesh (MP), West Bengal, Bihar, Odisha, Telangana, Assam, Jharkhand, and Bihar]. Samples fulfilling the inclusion criteria were packed in dry ice in triple-layer packaging, as per the International Air Transport Association (IATA) guidelines. The samples were subsequently transferred to the apex laboratory at ICMR-NIV, Pune, for molecular diagnosis, serology, and genomic analysis.

### Data Collection From Patients

All samples were accompanied by a case report form (CRF) of the patient, among which most of the forms were incomplete with respect to symptoms, comorbidities, clinical management, complications, and outcomes. Therefore, all cases found positive for ZIKV by qRT-PCR (*n* = 67) were interviewed telephonically in English, Hindi, and Malayalam language and the above details were elicited. The telephonic interviews also helped in validating the information captured in the CRF forms. The patients were surveyed for their demographic details, duration of illness, and clinical symptoms including details of hospitalization. Each call lasted for at least 10 min.

### Real-Time Reverse Transcriptase Polymerase Chain Reaction Testing

All 1,520 clinical samples received from the VRDLs at the apex laboratory at ICMR-NIV, Pune were screened for the presence of ZIKV, DENV, and CHIKV by CDC trioplex real-time RT-PCR assay (Santiago et al., 2018). Further, the Zika positive samples were screened with monoplex ZIKV real-time reverse transcriptase polymerase chain reaction (rRTPCR) (Lanciotti et al., 2008) and the quantification of the RNA copy numbers was performed by a standard curve generated using ZIKV *in vitro* transcribed RNA.

### Anti-Zika IgM Antibody Detection

The anti-Zika IgM antibodies were determined using ZIKV Detect IgM Capture Enzyme-linked Immunosorbent Assay (ELISA) (InBios International Inc., Seattle, WA, United States) as per the manufacturer’s instructions (In-Bios, 2017). For each sample, an immune status ratio (ISR) was calculated by dividing the ZIKV recombinant envelope (E) glycoprotein antigen absorbance (OD_450_) value by the cross-reactive control antigen OD_450_ value. ISR value > 1.90 indicates presumptive ZIKV positive.

### Anti-dengue IgM Antibody Detection

The anti-dengue IgM ELISA was performed by using ICMR-NIV dengue IgM capture ELISA kit in a subset of positive samples. Briefly, in prewashed anti-human IgM coated wells, 50 μl diluted serum samples (1:100) were added. The plate was incubated at 37°C for 1 h. In the subsequent step Dengue antigen (50 μl) was added into each well and incubated again at 37°C for 1 h. At the end of the incubation, 50 μl of avidin-HRP was added and incubated at 37°C for 1 h. Plates were washed five times after each incubation. The TMB substrate (100 μl) was added and the plate was incubated at room temperature for 10 min. After adding the stop solution, the optical density (OD) was measured at 450 nm. The OD value of less than 2.0 was considered as negative, and more than 3.0 as positive while OD values between upper and lower cut-off (2.0–3.0) were considered as equivocal. Respective positive and negative controls were used in the assay (Cecilia et al., 2011).

### Zika Virus Plaque Reduction Neutralization Test

The positive samples were further analyzed for the neutralizing antibody (NAb) titers by the plaque reduction neutralization test (PRNT90) as described earlier (Russell et al., 1967; Ward et al., 2018; Ribeiro et al., 2020). The presence of neutralizing antibodies was determined as described by Russell et al. with some modifications (Ribeiro et al., 2020). Vero CCL-81 cell suspension (1.0 × 10^5^/mL/well) was added into 24-well tissue culture plates and was incubated for 16–24 h at 37°C with 5% CO_2_ to obtain confluent monolayer. The serum samples were inactivated at a 56°C-water bath for 30 min. A ten-fold serial dilution of serum samples were mixed with an equal volume of virus suspension containing 50–60 plaque-forming units (PFU/0.1 ml) and incubated at 37°C for 1 h. The virus–serum mixtures (0.1 ml) were added onto the Vero CCL-81 cell monolayers and incubated at 37°C with 5% CO_2_ with intermittent shaking for uniform absorption of the virus. After 1 h, the suspension was aspirated, and the cell monolayer was gently overlaid with an overlay medium containing 2% carboxymethylcellulose (CMC) and 2X MEM containing 2% FBS (1:1). The plates were further incubated at 37°C with 5% CO_2_ for 4 days. The plates were terminated by decanting overlay medium followed by 0.1% saline wash and were stained with 1% amido black for 1 h at room temperature. The plates were washed in running tap water and air-dried. Plaque number was counted, and antibody titers are defined as a reciprocal of the highest dilution of tested serum that resulted in a reduction of viral infectivity by 90% when compared to the non-neutralization control (Russell et al., 1967; Ward et al., 2018).

### Next Generation Sequencing and Phylogenetic Analysis

Zika virus positive samples were sequenced using next generation sequencing (NGS) and the standard sanger sequencing method as described earlier (Yadav et al., 2018, 2020). Genomic characterization of the viral specimens was carried out using NGS with the quantified RNA. Briefly, RNA was extracted using Magmax RNA extraction kit (Thermo Fisher Scientific, United States), quantified by Qubit™ RNA high sensitivity (HS) kit (Thermo Fisher Scientific, United States) and the RNA libraries were prepared by using TruSeq stranded total RNA library preparation kit. Quantification of libraries was performed using Qubit™ dsDNA HS kit (Thermo Fisher Scientific, United States; kit and Qibitvrsion 2.0). Quantified libraries were pooled, normalized, and loaded on the Illumina Nextseq500/550 sequencing platform. Reference-based mapping was performed to retrieve the complete genome sequence of the virus isolates using the CLC Genome Workbench v20. Samples, where the complete genome could not be retrieved, were further tried for partial envelop [E] gene sequencing using the Sanger sequencing method. As described above, DENV positive samples having a cyclic threshold (Ct) value of < 30 were also sequenced using the TruSeq stranded total RNA library preparation kits and Miniseq sequencing platform. The complete genome sequences were retrieved using reference-based mapping with the DENV-I, II, III, and IV serotypes. A neighbor-joining (NJ) phylogenetic tree was constructed from the coding region of the ZIKV complete genome, partial E-gene, DENV1, DENV2, DENV3, and DENV4 separately using the Tamura 3-parameter model along with gamma distribution as the rate variation parameter. A bootstrap replication of 1,000 cycles was performed to assess the statistical robustness of the generated tree. The amino acid variation for each gene, as well as the net nucleotide and amino acid divergence were identified using the MEGA software version 7.0. The pairwise comparison was used to find the percentage identity for nucleotide and amino acid using CLC genomic workbench version 2.0.

### Partial E Gene Sequencing

For the partial ZIKV E gene amplification, the primer sets used were: Zika_F1555:AGGACAGGCCTTGACTTTTCAGAT and Zika_R_2350:GTGAGAACCAGGACATTCCTCC generating 795bp product. This was followed by a heminested PCR using Zika_F1555: AGGACAGGCCTTGACTTTTCAGAT and Zika_R_1950: TACTGTACCTCCACTGTGACTGT resulting in a 400bp product. The RT-PCR conditions followed for amplification were: reverse transcription at 50°C for 10 min, initial PCR activation at 98°C for 2 min, 35 amplification cycles of denaturation at 98°C for 10 s, annealing at 52°C for 10 s, extension at 72°C for 1 min. The PCR conditions for heminested PCR were: initial denaturation at 98°C for 2 min, 35 cycles of denaturation at 98°C for 10 s, annealing at 52°C for 30 s extension at 72°C for 30 s, and a final extension at 72°C for 5 min. The partial E gene was further sequenced by the sanger sequencing method and used for analysis as described earlier (Yadav et al., 2020).

## Results

### Viral Load in the Blood, Urine, and Serum Samples of the Zika Virus Cases and Other Arboviruses

After rRTPCR testing of 1,520 clinical samples, 75 samples from 67 patients were found to be positive for ZIKV, three of which were cases of co-infection. ZIKV and DENV co-infection was found in two cases while ZIKV, CHIKV, and DENV co-infection was observed in one case. Apart from this, samples from 121 patients were DENV positive while 10 were CHIKV positive. Dual positivity of DENV and CHIKV was observed in five cases. Serotyping of dengue was done and circulation of all four serotypes was seen. DENV-II was found to a larger extent in 26 patients followed by 7 cases of DENV-IV, 2 cases of DENV-I, and one case of DENV-III. Diagnosis of the DENV and CHIKV positive samples had been missed in initial screening at VRDLs as the primary test conducted there was NS1 and IgM ELISA for DENV and IgM ELISA for CHIKV. The ZIKV RNA log copy number ranged from 4.1 to 7.1 for plasma (*n* = 28) and urine (*n* = 10) samples, while 5.0–8.4 for serum samples (*n* = 29).

### Geographical Distribution of the Zika Virus Cases

**FIGURE 1:**
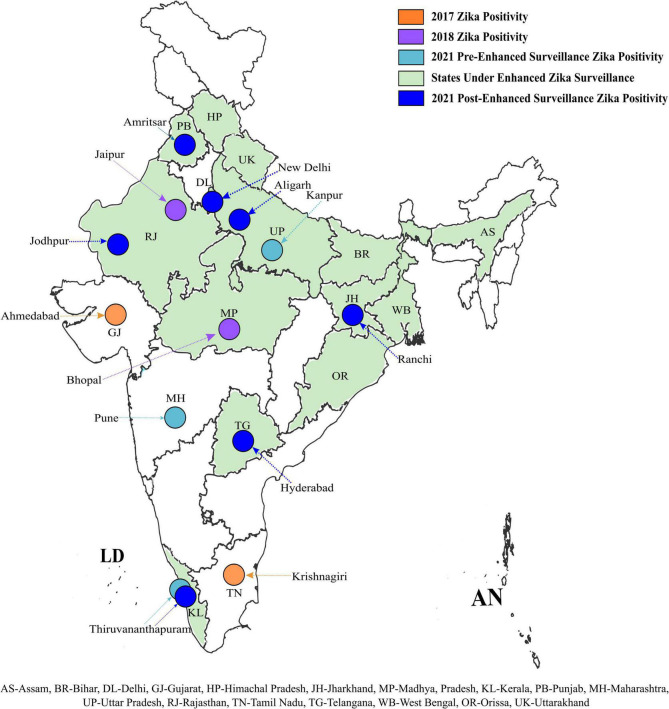
Geographical distribution of Zika positive cases in India: ZIKV positivity of reported during the earlier and current ZIKV surveillances in different states and UT of India. ZIKV positivity observed during different surveillance are marked in different colors. The bottom of the figure denotes the abbreviation of the states marked on the map. The outline of the Indian map was approved from survey of India and edited in Inkscape version 1.1.2.

The ZIKV positive samples detected during retrospective testing at ICMR-NIV, Pune revealed the presence of ZIKV in Amritsar, Punjab (1/120); New Delhi (1/64); Aligarh, UP (2/288); Jodhpur, Rajasthan (1/120); Ranchi, Jharkhand (1/120); Hyderabad, Telangana (1/60), and Thiruvananthapuram, Kerala (60/120), during 2021 (Table 1). This positivity was additional to the earlier reported positivity in the same year from Thiruvananthapuram, Kerala (166 cases) (unpublished data, Govt. of Kerala); Kanpur, UP (151 cases) (unpublished data, VRDL at King George Medical University, Lucknow); and Pune, Maharashtra (1 case). The retrospective analysis undertaken by us shows the spread of ZIKV to newer states and cities in India such as Delhi, Jharkhand, Punjab, Telangana, Jodhpur, and Aligarh. The ZIKV positivity so far reported in India is depicted in Figure 1.

**TABLE 1:** Positivity of Zika virus (ZIKV), dengue virus (DENV), and chikungunya virus (CHIKV) disease from different virus research and diagnostic laboratories (VRDLs) in India during May–October 2021.

States/UT	Name of the VRDL	Total cases screened	Positive by CDC trioplex real time RT-PCR assay					
			ZIKV	DENV	CHIKV	ZIKV + DENV	DENV + CHIKV	ZIKV + DENV + CHIKV
Uttarakhand	Government Medical College, Haldwani	18	0	2	0	0	0	0
Punjab	Government Medical College, Amritsar	120	0	10	5	0	0	**1**
Himachal Pradesh	Dr. RP Government Medical College, Tanda	51	0	0	0	0	0	0
New Delhi	All India Institute of Medical Sciences, New Delhi	63	0	0	0	0	2	0
	Dr. Ram Manohar Lohia Hospital, New Delhi	1	**1**	0	0	0	0	0
Uttar Pradesh	Jawaharlal Nehru Medical College, Aligarh, Uttar Pradesh	288	**1**	76		**1**	0	0
Rajasthan	All India Institute of Medical Sciences, Jodhpur, Rajasthan	120	**1**	16	0	0	**1**	0
Madhya Pradesh	ICMR-National Institute of Research in Tribal Health, Jabalpur, Madhya Pradesh	184	0	9	0	0	0	0
Jharkhand	Rajendra Institute of Medical Sciences, Ranchi, Jharkhand	120	**1**	0	0	0	0	0
West Bengal	ICMR- National Institute of Cholera and Enteric Diseases, Kolkata, West Bengal	36	0	0	0	0	0	0
Bihar	ICMR-Rajendra Memorial Research Institute of Medical Sciences, Patna, Bihar	84	0	1	0	0	0	0
Odisha	ICMR-Regional Medical Research Centre, Bhubaneswar, Odisha	60	0	2	0	0	0	0
Assam	ICMR-Regional Medical Research Centre, Dibrugarh, Assam	73	0	0	0	0	0	0
Telangana	Gandhi Medical College, Secunderabad, Telangana	60	0	2	0	0	2	0
	Osmania Medical College Hyderabad, Telangana	60	**1**	1	0	0	0	0
Kerala	Government Medical College, Thiruvananthapuram, Kerala	137	**59**	2	5	1	0	0
Total		1475	**64**	121	10	**02**	05	**01**

*The bold value represent positive Zika cases.*

### Clinical Presentation

**FIGURE 2:**
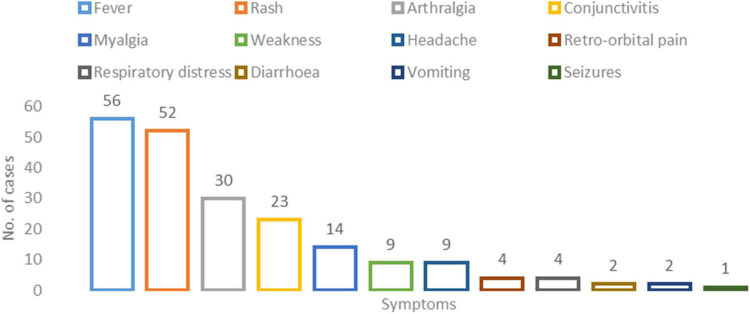
Clinical profile of the Zika virus positive cases: *X*-axis represents the number of cases per symptom and *Y*-axis represents the symptom type.

Among the 67 ZIKV positive cases, 13.43% (Rocha et al., 2019) were hospitalized while 86.56% (58) of the cases were managed on an outpatient basis. The median age and the interquartile range of the patients included in the study were 30 years (22−43 years). A total of 34 (50.7%) of ZIKV-positive cases were males. All 67 patients were symptomatic with fever 56 (83.6%) and rash 52 (77.6%) being the most prominent. Followed by arthralgia 30 (44.9%) and conjunctivitis 23 (34.3%). Microcephaly was reported in one patient. The median duration of illness amongst all cases was 6 days (range 2 to 10 days) (Figure 2 and Supplementary Table 4).

Three cases of co-infection of ZIKV with other flaviviruses were confirmed by qRT-PCR. One case each from Aligarh, UP, and Thiruvananthapuram, Kerala were confirmed for DENV and ZIKV positivity by qRT-PCR assay while one case (51 years/M) from Amritsar, Punjab was confirmed to have co-infection with all three, namely, DENV, ZIKV, and CHIKV. There was no significant difference in the profile of clinical symptoms and duration of illness between the patients suffering from standalone ZIKV and those with co-infection (CHIKV and DENV).

Six cases presented with atypical clinical presentation as compared to the rest. This included respiratory distress (*n* = 4 cases), seizure (*n* = 1 case), and suspected microcephaly at birth (*n* = 1 baby) related to ZIKV infection and of them three cases were hospitalized. Details of the cases are as follows:

***(1) Four cases with respiratory distress***—Four ZIKV positive cases from Rajasthan (24/M; case 1), Telangana (21/F; case 2), and Kerala (88/M; case 3 and 33/M; case 4) states presented with the complaints of respiratory distress screened negative for SARS-CoV-2 and Influenza. Their fever lasted for an average duration of 4–5 days. Only case 3 was hospitalized in August 2021 with complaints of fever and loss of appetite. He was known hypertensive and asthmatic. The illness resolved uneventfully in 4 days and the patient was discharged. Cases 2, 3, and 4 presented with respiratory distress, high-grade fever, and arthralgia. Cases 1 and 2 additionally reported vomiting and rash. None of the patients required supplemental oxygen or ventilation. The respiratory distress resolved within 4–5 days.

***(2) A Case with seizure***—A 45-year-old female presented with acute onset fever, arthralgia, conjunctival congestion, maculopapular rash, and convulsions in a tertiary care hospital in Thiruvananthapuram, Kerala. She had a previous history of seizures in 2002 after sustaining a traumatic head injury in a road accident. She was not on any anti-epileptic medication for the past several years. At the time of admission, her MRI brain was normal while EEG was abnormal with an intermittent sharp activity which was non-generalizable and focused in the left temporal region. As a management for seizures, she was given the anti-epileptic drug lacosamide (100 mg BD). She tested positive for ZIKV and was discharged post-recovery after 5 days of admission. She was asked to continue the anti-epileptic medications and no sequel has been observed during the follow-up visit in February 2022.

***(3) A case of suspected microcephaly at birth***—From Thiruvananthapuram, Kerala, a preterm (35 weeks + 5 days) baby girl weighing 1.68 kg (small for gestational age) was delivered in July 2021 by cesarean section due to placenta praevia. The baby was a suspect case of microcephaly as her head circumference at birth was 28 cm. According to Indian Standards for Fetal biometry head circumference at 35 weeks is 30 cm (Warrier and Ashokan, 2016). Her APGAR score was normal and there was no neurological deficit, seizure, or limb weakness. No visual or hearing impairment or any other signs of congenital zika syndrome were recorded (Freitas et al., 2020). Within 2 days of birth, she was diagnosed with congenital broncho-pneumonia and neonatal jaundice. The baby was intubated and started on empirical treatment with broad-spectrum antibiotics. Her serum samples were positive for ZIKV RNA by qRT-PCR and negative for TORCH pathogens (Toxoplasma, Rubella, Cytomegalovirus, Herpes Simplex). Serum samples of her mother were ZIKV negative by qRT-PCR, suggestive of probable infection resolution after initial transient viremia. The baby recovered after 12 days of birth and was discharged with advice to further follow up. She was regularly followed up for the gross motor, fine motor reflexes, head and chest circumference, length, weight gain, and neurological development. She gained normal head circumference as per the Indian Academy of Pediatrics growth chart (37 cm/40.5 cm) post 3/6 months of birth. During her last follow-up at 6 months of age, she had shown good weight gain, normal head circumference (40.5 cm), no neurological, ocular, auditory problems, or any developmental milestone delays.

### Anti-Zika Virus IgM and Plaque Reduction Neutralization Test 90 Neutralizing Antibody Responses

Out of 29 ZIKV positive samples from different patients, four samples showed the presence of anti-Zika IgM antibodies [Thiruvananthapuram (*n* = 3) and Aligarh (*n* = 1)]. The anti-Zika IgM positive samples were also checked for flavivirus cross-reactivity and one sample was found to be positive for anti-dengue antibodies while the remaining three were negative by ELISA. In addition, the Zika PRNT90 assay confirmed the presence of Zika-specific antibodies in all four samples.

### Next Generation Sequencing and Phylogenetic Analysis on the Zika Virus and Dengue Virus Positive Samples

**FIGURE 3:**
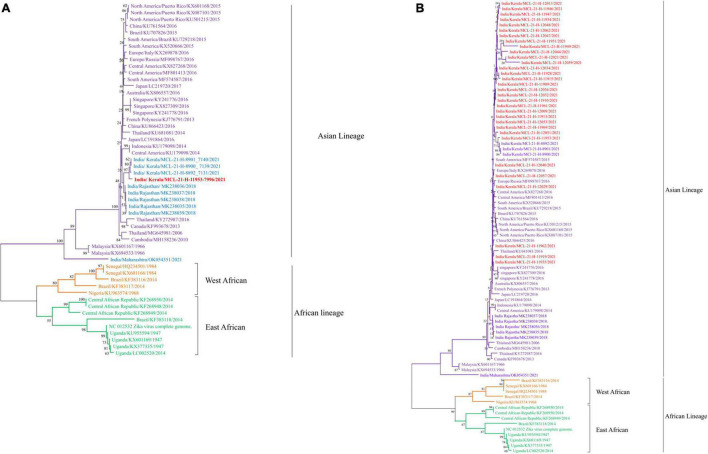
**(A,B)** The Phylogenetic tree constructed with Neighbor Joining (NJ) method for ZIKV genome sequence using the Tamura 3 as substitution model and a bootstrap replication of 1000 cycles. **(A)** ZIKV complete genome sequence; **(B)** ZIKV partial envelope gene sequence.

Next generation sequencing was performed on all the ZIKV, DENV, and CHIKV positive samples, for identifying the currently circulating ZIKV, DENV, and CHIKV strains in various parts of the Indian population. The analysis involved generating a consensus sequence from all sequence reads for each sample using CLC genomic software version 21.0.4. Out of the 67 ZIKV positive cases [Ct value range—29–33], the partial fragment of the E gene was sequenced for 32 ZIKV positive cases while one complete genome was retrieved (Figures 3A,B). To better understand the relationship between these sequences, we used the envelope coding region to construct the phylogenetic analysis between the ZIKV strains (Figure 3B). The phylogenetic tree shown in Figure 3B indicated that ZIKV sequences obtained in the current study belonged to the Asian lineage and were closely related to the strain previously reported from India (Yadav et al., 2019; Malhotra et al., 2020). The percent identities were calculated in CLC Genomics workbench version 22.0. The identities observed were 89%, 95.53%, and 99.67% at the nucleotide level and 96.75%, 96.99%, and 99.397% at amino acid (AA) level (Supplementary Table 1) between the complete genome of ZIKV obtained in this study and the ZIKV sequences from Uganda (NC_012532), Kerala (OM666892), and Rajasthan (MK238036), respectively (Figure 3A). The comparison at the nucleotide and AA level was performed for the partial envelope gene with the sequences from Kerala and Rajasthan and it indicated 97%–100% and 95%–99%, and 96%–100% and 92%–100% identity, respectively (Figure 3B and Supplementary Table 1). All the ZIKV sequences from the current study belong to Asian lineage (Figures 3A,B). The ZIKV sequences in the current study sequences were matched with the reference genome of ZIKV (NC_012532) from Uganda. Whereas the 32 ZIKV partial sequences showed an average of 84% nucleotide and 91% amino acid identities with the reference genome of ZIKV (NC_012532) from Uganda (Supplementary Table 1). The amino acid changes observed are depicted in Supplementary Figure 1. The point mutation in prM-S139N reported amongst the children with microcephaly was not observed in the retrieved ZIKV sequences. The NS1-A188V mutation reported to enhance ZIKV infectivity in the laboratory *Aedes aegypti* mosquitoes was also not observed in the retrieved sequence (Liu et al., 2017). The genomic data retrieved in the current study are provided in Supplementary Table 3.

**FIGURE 4:**
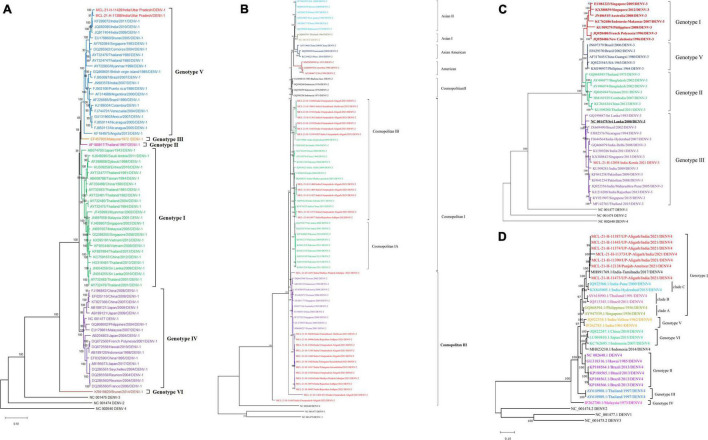
**(A–D)** The phylogenetic tree constructed with Neighbor Joining (NJ) method for DENV using the Tamura 3 as substitution model and a bootstrap replication of 1000 cycles. **(A)** DENV-1 serotype; **(B)** DENV-2 serotype; **(C)** DENV-3 serotype, and **(D)** DENV-4 serotype.

Out of 129 DENV positive samples, sequences from 36 serum samples were retrieved and analyzed with neighbor-joining phylogenetic method using Tamura-3 as substitution model and a bootstrap replication of 1,000 cycles. The analysis indicated the circulation of DENV-1 (*n* = 2), DENV-2 (*n* = 26), DENV-3 (*n* = 1), and DENV-4 (*n* = 7) serotypes in different geographical locations of the country. The genomic data retrieved for the DENV (1-4) (Dick, 1952; Moore et al., 1975; Rothan et al., 2018; Pielnaa et al., 2020) is given in Supplementary Table 2. The presence of the DENV-2 serotype was confirmed in Haldwani, Aligarh, Jodhpur, Jabalpur, and Secunderabad districts. The DENV-4 serotype was seen in Aligarh and Punjab while the DENV-III serotype was confirmed from Thiruvananthapuram. The co-circulation of all the three DENV-1, DENV-2, and DENV-4 serotypes was seen in Aligarh. The phylogenetic analysis of the two sequences of DENV-1 serotype showed alignment with genotype V of the Indian sequence. Furthermore, 26 sequences of the DENV-2 formed two distinct genotypes [Cosmopolitan I (*n* = 12) and Cosmopolitan III (*n* = 14)] having similarities with the sequences from Pakistan, Sri Lanka, India, and China. One sequence of DENV-3 clustered with sequences from India, Pakistan, Brazil, Singapore, and Thailand which belonged to genotype III. While seven sequences of the DENV-4 clustered with sequences from Tamil Nadu and belonged to genotype I (Figures 4A–D). Of the CHIKV positive samples, the Ct range was higher (Asadi-Pooya, 2016; Warrier and Ashokan, 2016; Landry and St George, 2017; Lina Villa et al., 2017; Liu et al., 2017; Ward et al., 2018; Yadav et al., 2018, 2020; Freitas et al., 2020; Malhotra et al., 2020; Silva et al., 2020; Low et al., 2021; Stiasny et al., 2021) and NGS could not lead to giving useful sequences to be used for analysis. The amino acid changes in the NS5 protein of DENV-1-DENV-4 observed with respect to its reference are depicted in Supplementary Figures 2, 5 respectively. The NS5 protein of DENV is nearly 900 peptide residue with multifunctional activity. The 1/3rd region toward the N terminal encoding for the methyltransferase and the rest toward the C terminal encodes for the RNA-dependent RNA Polymerase (RdRp). It is observed that the mutations are observed in both the segments of the protein. CHIKV genomic sequences were not retrieved from the real-time RT-PCR samples in the study; the reason being low volume and higher CT values of the samples.

## Discussion

Prior to this study, the different sentinel surveillance laboratories in our network reported ZIKV cases from Ahmadabad, Gujarat (2017), Tamil Nadu (2017), Jaipur, Rajasthan (2018), Bhopal (2018), Thiruvananthapuram, Kerala; Kanpur, UP, and Pune, Maharashtra before August 2021 (15–18). The number of cases reported in Kerala state and Uttar Pradesh during the year 2021 prior to this study, once again emphasized that active surveillance only can help in tracking a disease like ZVD to understand its impact on the health system (Yadav et al., 2022). The retrospective surveillance in this study indicated the spread of ZIKV to newer areas where it had never been reported earlier, thus establishing local transmission of ZIKV in India. Overall, from 2017 to 2021, the presence of ZIKV has now been reported in 16 states/UTs of India (Figure 1). In this study, from all the surveillance areas except Thiruvananthapuram, only 1–2 cases of ZIKV were detected. Considering this, the possibility of imported infections in the newer areas from the previously reported states cannot be ruled out. Same time COVID-19 pandemic when the whole health system focused much on fighting COVID-19 and its related aspect in a long tiring battle, the vector control was a compromise and huge rain in these states provided additional opportunities to enhance the breeding sites and mosquito population. However, during telephonic interviews of patients, no history of inter-state travel or contact with a ZIKV positive traveler could be elicited but the earlier establishment of this importation from one state to another cannot be ruled out. Of the 67 symptomatic positive cases detected in this study, the fever and rash were present in 84% and 78% of the cases, respectively. However, we also detected cases of respiratory distress, seizures, and suspected microcephaly as the clinical presentation of ZVD as reported earlier by Asadi-Pooya (2016) and Lina Villa et al. (2017). The ZIKV positive case who presented with seizures had a previous history of post-traumatic seizures almost two decades ago. The patient presented to the hospital with the complaint of seizure but also tested positive for ZIKV simultaneously. Though we could not conclusively establish a causal relationship between the infection and clinical manifestation, the possibility of ZIKV infection leading to seizures cannot be completely ruled out. The preterm baby with suspected microcephaly showed achievable milestones after initial complications and discharge from the neonatal intensive care unit. The head circumference became normal as per age and no developmental anomalies were seen. The microcephaly at birth could be linked with ZVD considering the ZIKV RNA positivity in serum. However, we could not establish a causal relationship with ZIKV infection as the baby’s mother tested negative by qRT-PCR.

We observed that during initial NS1 + IgM and IgM-based screening at VRDLs for DENV and CHIKV respectively, many positive cases (121 and 10 samples tested positive for DENV and CHIKV respectively) by the rRT-PCR were missed. Besides this, co-infections of DEN/CHIK, DEN/ZIKV, and DEN/CHIK/ZIKV were seen in 5, 2, and 1 patient, respectively by the rRT-PCR testing at ICMR-NIV. This indicates that the current algorithm of using ELISA as a frontline diagnostic test for flaviviruses in India needs to be re-looked and supplemented with rRT-PCR-based testing. Unlike other flaviviruses, serological testing for ZIKV is not considered to be a frontline diagnostic test due to the high cross-reactivity of ZIKV with other flaviviruses particularly dengue (Stiasny et al., 2021). We tested a subset (29 samples) of rRT-PCR positive ZIKV samples for the presence of IgM antibodies. The anti-ZIKV IgM antibodies were detected in 4/29 samples, of which, one was a case of co-infection of ZIKV and DENV. The ELISA IgM results of these four samples had 100% concordance with the results of the PRNT90 assay for ZIKV. However, these findings need to be validated on a larger sample size. All the four patients with IgM positivity had reported onset of illness within 4–5 days. This matches with the published literature where IgM antibodies for ZIKV have been reported as early as within 4 days of onset of illness (Landry and St George, 2017; Silva et al., 2020; Low et al., 2021). We could not test more positive or negative samples for ZIKV IgM and PRNT due to the limitation in sample volumes received from various VRDLs for retrospective screening. It would also be valuable to look at the presence of anti-ZIKV IgM antibodies in the rRT-PCR ZIKV negative samples considering the short viremia period of ZIKV. This would help in formulating appropriate algorithms for laboratory detection of ZIKV.

In addition to ZIKV, we also undertook serotyping and genotyping of circulating strains of DENV from the rRT-PCR positive samples. In line with the previous reports, we found circulation of all four dengue serotypes (DEN 1–4) (Silva et al., 2020). The predominant DENV genotypes reported in this study are G-V of DENV-1; cosmopolitan genotype of DENV-2; GIII of DENV-3, and G-I of DENV-4. These strains have been reported earlier in India (Kasirajan et al., 2019; Alagarasu et al., 2021).

In several ZIKV outbreaks, S139N substitution in the pre-membrane region and A188V/T233A mutations in the NS1 region of ZIKV has been implicated to enhance neurovirulence and increase the chances of microcephaly (Rossi et al., 2018; Wongsurawat et al., 2018). Also, the mutation in the envelope protein E-V473M was known to be responsible for increased virulence, and high maternal to fetal (vertical) transmission (Wongsurawat et al., 2018). However, Wongsurawat et al. had shown microcephaly in an aborted fetus of a mother infected with Asian lineage of ZIKV without S17N substitution (Wongsurawat et al., 2018). Considering this, a single substitution in the ZIKV genome is not a reliable marker for predicting neurovirulence/microcephaly. In this study, these mutations were not observed in the single whole genome sequence of ZIKV which was retrieved from the positive sample. The 32 partial envelope gene sequences retrieved in this study matched the sequences of the 2018 Rajasthan outbreaks and indicated circulation of the Asian lineage of ZIKV in India. Our earlier studies also report the circulation of two different ZIKV Asian sub-lineages in India. During the 2017 outbreak of Ahmadabad, Gujarat and 2021 outbreak of Pune, Maharashtra, we found the ZIKV strains matching with the 1966 Malaysia strains isolated from *Aedes* sp. (Yadav et al., 2019). Later, the ZIKV strains sequenced during the 2018 Rajasthan outbreak clustered separately as compared to the Gujarat strains (Sapkal et al., 2018; Yadav et al., 2019).

The retrospective surveillance for ZIKV undertaken by us demonstrates the silent spread of this virus to almost all parts of India with a predominance of the more recent 2018 Rajasthan ZIKV strain. Our results indicated the need for continuous and enhanced surveillance for ZIKV along with DENV and CHIKV with emphasis on the ante-natal ZIKV screening. It is also critical to strengthen linkages of ZIKV surveillance sites with the existing newborn birth defect screening sites in the country to understand the spectrum of ZVD in babies born to ZIKV infected mothers. Development of quick and reliable tests as well as validating the utility of simple serology-based tests for ZIKV will help in augmenting the diagnostic capabilities. With the massive upscaling of the COVID-19 rRT-PCR testing laboratories in India, this network can also be re-purposed for augmenting ZIKV testing in the country. Along with these efforts, it is also essential not to lose sight of effective vector control measures and focus on the development of a safe and effective vaccine for ZIKV, which could be administered to pregnant women.

## Data Availability Statement

The datasets presented in this study can be found in online repositories. The names of the repository/repositories and accession number(s) can be found in the article/Supplementary Material.

## Ethics Statement

The studies involving human participants were reviewed and approved by the Institutional Human Ethics Committee at ICMR-National Institute of Virology Pune India [NIV/IEC/March/2021/D-9 dated April 2021]. Written informed consent to participate in this study was provided by the participants’ legal guardian/next of kin.

## Author Contributions

PY and NG did the conceptualization. PY, NG, HK, RS, GNS, GD, AS, TM, SP, PG, JY, KK, AK, PP, MD, and YJ performed the methodology. AK, SP, PP, DN, YJ, and MD carried out the software. KS, SMa, HS, HMK, AM, KD, RG, VS, MS, ND, MC, LD, RA, KN, BB, GS, IP, SD, PB, SJ, and VR carried out the resources. PY, NG, HK, RS, AS, AK, and GD wrote the original draft of the manuscript. PY, NG, HK, RS, AS, GD, and GNS supervised the data. PY, NG, HK, and GNS carried out the project administration. PY carried out the funding acquisition. All authors have read and agreed to the published version of the manuscript.

## Conflict of Interest

The authors declare that the research was conducted in the absence of any commercial or financial relationships that could be construed as a potential conflict of interest.

## Publisher’s Note

All claims expressed in this article are solely those of the authors and do not necessarily represent those of their affiliated organizations, or those of the publisher, the editors and the reviewers. Any product that may be evaluated in this article, or claim that may be made by its manufacturer, is not guaranteed or endorsed by the publisher.

## Supplementary Material

Supplementary Figure 1 Changes in the amino acid sequences of Zika virus with respect to reference: NC 012532.

Supplementary Figure 2 Changes in the amino acid sequences of Dengue virus-1 in the NS5 gene retrieved in the study with respect to reference NC_001477.

Supplementary Figure 3 Changes in the amino acid sequences of Dengue virus-2 in the NS5 gene retrieved in the study with respect to reference NC_001474.

Supplementary Figure 4 Changes in the amino acid sequences of Dengue virus-3 in the NS5 gene retrieved in the study with respect to reference NC_001475.

Supplementary Figure 5 Changes in the amino acid sequences of Dengue virus-4 in the NS5 gene retrieved in the study with respect to reference NC_002640.1.

Supplementary Table 1 The amino acid and nucleotide identities percentage of enveloped gene (E) and full genome of ZIKV retrieved in this study with different reference genomes.

Supplementary Table 2 Percent nucleotide and amino acid identities of the Dengue sequences.

Supplementary Table 3 Genomic Data of the ZIKV partial (E-gene), ZIKV Complete sequences, Dengue Serotypes analyzed in the study.

Supplementary Table 4 Clinical Profile of Zika positive cases (*n* = 67).
