# Supplementary material for: Zika a Vector Borne Disease Detected in Newer States of India Amidst the COVID-19 Pandemic
Source: Front Microbiol. 2022 Jun 10;13:888195. doi: 10.3389/fmicb.2022.888195 (PMC9226610; doi:10.3389/fmicb.2022.888195)

Supplementary Table-1: The Percentage amino acid and nucleotide identities of enveloped gene (E) & full genome of ZIKV retrieved in this study with different reference genomes

|  | **Length** | | **Zika virus reference strain (MR766**  **NC_012532)yp** | | **ZIKV Asian strain (Rajasthan outbreak 2018_** **MK238036)** | | **ZIKV Asian strain (Kerala 2021_OM666892)** | |
| --- | --- | --- | --- | --- | --- | --- | --- | --- |
| **Current study** | Gene (nucleotide) | Protein (amino acid) | PNS | PAS | PNS | PAS | PNS | PAS |
| MCL-21-H-11953 (Complete genome) Accession: OM666891 | 10749 | 3583 | 88.44 | 96.75 | 95.53 | 96.99 | 99.67 | 99.97 |
| MCL-21-H-11953 (E gene) | 1448 | 483 | 83.46 | 88.68 | 99.26 | 99.23 | 99.76 | 100.00 |
| MCL-21-H-11909 (E gene) | 416 | 139 | 84.72 | 91.47 | 98.74 | 97.59 | 99.26 | 98.45 |
| MCL-21-H-12032 (E gene) | 414 | 138 | 84.13 | 90.50 | 98.99 | 98.41 | 99.51 | 99.23 |
| MCL-21-H-12036 (E gene) | 414 | 138 | 84.13 | 90.50 | 98.99 | 98.41 | 99.51 | 99.23 |
| MCL-21-H-12013 (E gene) | 418 | 139 | 83.85 | 89.59 | 98.75 | 97.61 | 99.26 | 98.46 |
| MCL-21-H-11910 (E gene) | 412 | 137 | 84.41 | 90.41 | 98.98 | 98.40 | 99.50 | 99.23 |
| MCL-21-H-11934 (E gene) | 418 | 139 | 84.34 | 90.58 | 99.00 | 98.43 | 99.51 | 99.24 |
| MCL-21-H-11961 (E gene) | 418 | 139 | 84.72 | 91.32 | 99.00 | 98.39 | 99.51 | 99.22 |
| MCL-21-H-12034 (E gene) | 418 | 139 | 84.78 | 91.47 | 98.74 | 97.59 | 99.26 | 98.45 |
| MCL-21-H-12048 (E gene) | 414 | 138 | 84.07 | 90.41 | 98.99 | 98.40 | 99.51 | 99.23 |
| MCL-21-H-12053 (E gene) | 412 | 137 | 84.51 | 90.41 | 98.99 | 98.40 | 99.51 | 99.23 |
| MCL-21-H-11928 (E gene) | 418 | 139 | 84.78 | 91.54 | 98.74 | 97.61 | 99.26 | 98.46 |
| MCL-21-H-12047 (E gene) | 407 | 136 | 84.19 | 91.24 | 98.97 | 98.37 | 99.50 | 99.22 |
| MCL-21-H-12062 (E gene) | 405 | 135 | 84.14 | 91.32 | 98.97 | 98.39 | 99.50 | 99.22 |
| MCL-21-H-11913 (E gene) | 395 | 132 | 83.56 | 89.98 | 98.94 | 98.33 | 99.48 | 99.20 |
| MCL-21-H-12040 (E gene) | 402 | 134 | 84.48 | 91.17 | 98.96 | 98.36 | 99.49 | 99.21 |
| MCL-21-H-11969 (E gene) | 391 | 130 | 83.33 | 90.93 | 98.93 | 99.17 | 99.48 | 100.00 |
| MCL-21-H-12051 (E gene) | 390 | 130 | 83.46 | 88.71 | 98.66 | 97.43 | 99.21 | 98.35 |
| MCL-21-H-12057 (E gene) | 389 | 130 | 84.79 | 90.84 | 98.65 | 97.43 | 99.21 | 98.35 |
| MCL-21-H-12059 (E gene) | 420 | 140 | 81.65 | 86.60 | 96.64 | 95.08 | 97.23 | 96.05 |
| MCL-21-H-11962 (E gene) | 369 | 123 | 84.67 | 91.24 | 98.57 | 97.24 | 99.16 | 98.23 |
| MCL-21-H-11919 (E gene) | 354 | 118 | 84.81 | 94.34 | 99.12 | 99.10 | 99.71 | 100.00 |
| MCL-21-H-11946 (E gene) | 355 | 118 | 84.88 | 94.34 | 99.12 | 99.10 | 99.72 | 100.00 |
| MCL-21-H-11947 (E gene) | 355 | 118 | 84.88 | 94.34 | 99.12 | 99.10 | 99.72 | 100.00 |
| MCL-21-H-12009 (E gene) | 357 | 119 | 83.42 | 89.82 | 98.83 | 98.13 | 99.72 | 99.10 |
| MCL-21-H-11915 (E gene) | 357 | 119 | 84.13 | 93.37 | 98.83 | 99.10 | 99.43 | 100.00 |
| MCL-21-H-11935 (E gene) | 349 | 116 | 85.08 | 94.24 | 99.11 | 99.08 | 99.71 | 100.00 |
| MCL-21-H-12029 (E gene) | 350 | 117 | 83.55 | 90.79 | 98.80 | 98.10 | 99.71 | 99.09 |
| MCL-21-H-11951 (E gene) | 358 | 119 | 81.21 | 88.40 | 97.59 | 97.10 | 98.55 | 98.15 |
| MCL-21-H-11949 (E gene) | 254 | 85 | 80.86 | 94.81 | 96.07 | 94.60 | 97.07 | 96.18 |
| MCL-21-H-12044 (E gene) | 278 | 93 | 81.55 | 91.21 | 97.28 | 95.06 | 98.51 | 96.49 |
| MCL-21-H-12021 (E gene) | 216 | 72 | 80.64 | 97.06 | 97.49 | 96.92 | 99.06 | 98.56 |

PNS- Percentage Nucleotide Similarity

PAS- Percentage Amino-acid Similarity

**Supplementary Table-**2: Percent nucleotide and amino acid identities of the Dengue sequences

| **Dengue Virus Serotype-1** | | | | | | | | | | | | | | | | | | | | | | | | |
| --- | --- | --- | --- | --- | --- | --- | --- | --- | --- | --- | --- | --- | --- | --- | --- | --- | --- | --- | --- | --- | --- | --- | --- | --- |
| **Sample ID/**  **Accession ID** | **DENV-1 Length (10735 bp)** | | | **Dengue virus -1 reference strain (NC_001477)** | | | | | **DENV-1**  **Asian strain**  **( Madhya Pradesh 2011_KF289072)** | | | | | | | | | | **DENV-1**  **Asian strain**  **(Singapore 1993_ AY762084)** | | | | | |
| **Current study** | Gene (nucleotide) | | Protein (amino acid) | PNI | PAI | | | | PNI | | | | PAI | | | | | | PNI | | | | PAI | |
| MCL-21-H-11428/  OM666557 | 10708 | | 3569 | 92.00 | 92.95 | | | | 98.60 | | | | 98.80 | | | | | | 96.95 | | | | 97.15 | |
| MCL-21-H-11388/  OM666558 | 10727 | | 3576 | 92.19 | 92.92 | | | | 98.92 | | | | 99.26 | | | | | | 97.17 | | | | 97.54 | |
| **Dengue Virus Serotype-2** | | | | | | | | | | | | | | | | | | | | | | | | |
| **Sample ID/**  **Accession ID** | **DENV-2 Length**  **(10670 bp)** | | | **Dengue virus-2 reference strain (NC_001474)** | | | | | | **DENV-2**  **Asian strain**  **( Hyderabad-2009_JX475906)** | | | | | | | **DENV-2**  **Asian strain**  **( Sri Lanka-1996_FJ882602)** | | | | | | | |
| **Current study** | Gene (nucleotide) | | Protein (amino acid) | PNI | | PAI | | | | PNI | | | | PAI | | | | PNI | | | PAI | | | |
| MCL-21-H-10600/  OM639979 | 10677 | | 3559 | 92.29 | | 79.71 | | | | 93.50 | | | | 82.77 | | | 93.85 | | | | 83.82 | | | |
| MCL-21-H-10947/  OM639980 | 10706 | | 3569 | 92.43 | | 80.13 | | | | 93.41 | | | | 82.64 | | | 93.78 | | | | 83.69 | | | |
| MCL-21-H-11017/  OM639981 | 10707 | | 3569 | 92.45 | | 80.03 | | | | 93.44 | | | | 82.68 | | | 93.81 | | | | 83.78 | | | |
| MCL-21-H-11025/  OM639982 | 10730 | | 3577 | 92.44 | | 80.03 | | | | 93.44 | | | | 82.68 | | | 93.82 | | | | 83.78 | | | |
| MCL-21-H-11361/  OM639983 | 10699 | | 3566 | 92.53 | | 80.72 | | | | 95.69 | | | | 88.65 | | | 96.30 | | | | 90.24 | | | |
| MCL-21-H-11365/  OM639984 | 10674 | | 3558 | 92.39 | | 79.90 | | | | 93.46 | | | | 82.61 | | | 93.75 | | | | 83.50 | | | |
| MCL-21-H-11373/  OM639985 | 10714 | | 3571 | 92.24 | | 79.71 | | | | 95.62 | | | | 88.58 | | | 96.14 | | | | 90.24 | | | |
| MCL-21-H-11381/  OM639986 | 10724 | | 3575 | 92.63 | | 80.88 | | | | 95.89 | | | | 89.07 | | | 96.49 | | | | 90.57 | | | |
| MCL-21-H-11392/  OM639987 | 10711 | | 3570 | 92.75 | | 81.14 | | | | 98.68 | | | | 96.77 | | | 96.77 | | | | 91.45 | | | |
| MCL-21-H-11399/  OM639988 | 10709 | | 3570 | 92.77 | | 81.11 | | | | 98.69 | | | | 96.80 | | | 96.78 | | | | 91.48 | | | |
| MCL-21-H-11403/  OM639989 | 10712 | | 3571 | 92.76 | | 81.11 | | | | 98.68 | | | | 96.74 | | | 96.77 | | | | 91.42 | | | |
| MCL-21-H-11405/  OM639990 | 10709 | | 3570 | 92.69 | | 80.82 | | | | 95.89 | | | | 89.04 | | | 96.47 | | | | 90.83 | | | |
| MCL-21-H-11427/  OM639991 | 10728 | | 3576 | 92.66 | | 80.84 | | | | 95.87 | | | | 89.13 | | | 96.48 | | | | 90.86 | | | |
| MCL-21-H-11449/  OM639992 | 10733 | | 3578 | 92.34 | | 80.03 | | | | 93.49 | | | | 83.00 | | | 93.86 | | | | 83.85 | | | |
| MCL-21-H-11634/  OM639993 | 10561 | | 3520 | 92.37 | | 79.89 | | | | 93.45 | | | | 82.56 | | | 93.76 | | | | 83.51 | | | |
| MCL-21-H-10601/  OM648092 | 10712 | | 3571 | 92.55 | | 80.75 | | | | 95.83 | | | | 88.91 | | | 96.43 | | | | 90.41 | | | |
| MCL-21-H-10906 | 10267 | | 3422 | 92.28 | | 79.44 | | | | 93.53 | | | | 82.59 | | | 93.79 | | | | 83.44 | | | |
| MCL-21-H-10974/  OM700180 | 10548 | | 3516 | 92.37 | | 80.01 | | | | 93.42 | | | | 82.68 | | | 93.77 | | | | 83.71 | | | |
| MCL-21-H-10977/  OM700181 | 10608 | | 3536 | 92.44 | | 80.33 | | | | 95.84 | | | | 88.98 | | | 96.38 | | | | 90.72 | | | |
| MCL-21-H-11204/  OM730078 | 10568 | | 3523 | 92.35 | | 79.70 | | | | 93.52 | | | | 82.77 | | | 93.82 | | | | 83.76 | | | |
| MCL-21-H-11369/  OM680963 | 10741 | | 3580 | 92.42 | | 79.97 | | | | 93.44 | | | | 82.84 | | | 93.79 | | | | 83.72 | | | |
| MCL-21-H-11400/  OM698821 | 10719 | | 3573 | 92.69 | | 80.84 | | | | 95.90 | | | | 89.13 | | | 96.48 | | | | 90.83 | | | |
| MCL-21-H-11408/  OM681318 | 10733 | | 3578 | 92.59 | | 80.88 | | | | 95.86 | | | | 89.04 | | | 96.49 | | | | 90.57 | | | |
| MCL-21-H-11428/  OM744110 | 10466 | | 3489 | 87.18 | | 71.47 | | | | 89.44 | | | | 77.36 | | | 89.73 | | | | 78.16 | | | |
| MCL-21-H-11443 | 10236 | | 3412 | 84.76 | | 66.30 | | | | 85.43 | | | | 67.88 | | | 85.64 | | | | 68.36 | | | |
| MCL-21-H-11653 | 9687 | | 3229 | 92.60 | | 80.53 | | | | 94.50 | | | | 85.32 | | | 94.83 | | | | 86.32 | | | |
| **Dengue Virus Serotype-3** | | | | | | | | | | | | | | | | | | | | | | | | |
| **Sample ID/**  **Accession ID** | **DENV3**  **(Length-10707 bp)** | | | **Dengue virus-3 reference strain (NC_001475)** | | | | | | | **DENV-3**  **Asian strain**  **( India_2009**  **_** **KU509281 )** | | | | | | | **DENV-3**  **Asian strain**  **( Singapore-2013_** **KX380842)** | | | | | | |
| **Current study** | Gene (nucleotide) | | Protein (amino acid) | PNI | | | PAI | | | | PNI | | | | PAI | | | PNI | | | | PAI | | |
| MCL-21-H-12058/  OM638675 | 10703 | | 3568 | 95.94 | | | 98.64 | | | | 97.67 | | | | 98.83 | | | 99.06 | | | | 99.58 | | |
| **Dengue Virus Serotype-4** | | | | | | | | | | | | | | | | | | | | | | | | |
| **Sample ID/**  **Accession ID** | | **DENV-4**  **(Length-10649 bp)** | | **Dengue virus-4 reference strain (NC_002640.1)** | | | | | | | | **DENV-4**  **Asian strain**  **( Tamil Nadu-2017_MH891769.1)** | | | | | | | | **DENV-4 Asian strain**  **( Thailand-1991_** **AY618990.1)** | | | | |
| **Current study** | | Gene (nucleotide) | Protein (amino acid) | PNI | | | | PAI | | | | PNI | | | | PAI | | | | PNI | | | | PAI |
| MCL-21-H-11390/  OM639994 | | 10637 | 3546 | 92.06 | | | | 90.53 | | | | 99.32 | | | | 97.73 | | | | 94.63 | | | | 95.45 |
| MCL-21-H-11373/  OM639995 | | 10649 | 3550 | 89.64 | | | | 68.09 | | | | 96.54 | | | | 71.59 | | | | 92.15 | | | | 69.32 |
| MCL-21-H-11374/  OM639996 | | 10620 | 3540 | 92.07 | | | | 89.47 | | | | 99.38 | | | | 98.86 | | | | 94.60 | | | | 94.32 |
| MCL-21-H-11387/  OM639997 | | 10608 | 3536 | 92.05 | | | | 89.47 | | | | 99.38 | | | | 98.86 | | | | 94.58 | | | | 94.32 |
| MCL-21-H-11443/  OM639998 | | 10700 | 3567 | 91.95 | | | | 89.47 | | | | 99.37 | | | | 98.86 | | | | 94.57 | | | | 94.32 |
| MCL-21-H-12138/  OM639999 | | 10687 | 3562 | 91.94 | | | | 88.42 | | | | 99.43 | | | | 97.73 | | | | 94.61 | | | | 93.18 |
| MCL-21-H-11473/  OM640000 | | 10516 | 3505 | 91.95 | | | | 89.47 | | | | 99.03 | | | | 98.86 | | | | 94.51 | | | | 94.32 |
| MCL-21-H-11390/  OM639994 | | 10637 | 3546 | 92.06 | | | | 90.53 | | | | 99.32 | | | | 97.73 | | | | 94.63 | | | | 95.45 |
| MCL-21-H-11373/  OM639995 | | 10649 | 3550 | 89.64 | | | | 68.09 | | | | 96.54 | | | | 71.59 | | | | 92.15 | | | | 69.32 |

**Supplementary Table-3: Genomic Data of the ZIKV partial (E-gene), ZIKV Complete sequences, Dengue Serotypes analyzed in the study**

| **Sequence ID** | **Real Time RT-PCR Ct values** | **Total reads** | **Relevant reads** | **Length (bp)** | **Percentage of Genome Retrival** | **Virus strain** |
| --- | --- | --- | --- | --- | --- | --- |
| MCL-21-H-11428 | Positive (15.92/15.10) | 660242 | 561808 | 10708 | 99.75 | Dengue-1 |
| MCL-21-H-11388 | Positive (19.03/19.26) | 670652 | 110306 | 10727 | 99.93 | Dengue-1 |
| MCL-21-H-10600 | Positive (Ct Value 24.38/24.18) | 247072 | 7554 | 10677 | 99.57 | Dengue-2 |
| MCL-21-H-10947 | Positive (18.67/18.27) | 76622 | 20968 | 10706 | 99.84 | Dengue-2 |
| MCL-21-H-11017 | Positive (15.53/15.02) | 731748 | 356258 | 10707 | 99.85 | Dengue-2 |
| MCL-21-H-11025 | Positive (13.99/14.00) | 5511620 | 5349321 | 10730 | 100.07 | Dengue-2 |
| MCL-21-H-11361 | Positive (22.71/22.55) | 65814 | 5188 | 10699 | 99.78 | Dengue-2 |
| MCL-21-H-11365 | Positive (21.74/21.65) | 162826 | 2235 | 10674 | 99.54 | Dengue-2 |
| MCL-21-H-11373 | Positive (19.27/19.20) | 207510 | 65137 | 10714 | 99.92 | Dengue-2 |
| MCL-21-H-11381 | Positive (17.12/17.10) | 291898 | 263393 | 10724 | 100.01 | Dengue-2 |
| MCL-21-H-11392 | Positive (22.74/22.62) | 195950 | 65364 | 10711 | 99.89 | Dengue-2 |
| MCL-21-H-11399 | Positive (20.34/20.28) | 170856 | 77898 | 10709 | 99.87 | Dengue-2 |
| MCL-21-H-11403 | Positive (21.81/21.70) | 133420 | 71451 | 10712 | 99.90 | Dengue-2 |
| MCL-21-H-11405 | Positive (21.27/21.21) | 200788 | 60412 | 10709 | 99.87 | Dengue-2 |
| MCL-21-H-11427 | Positive (14.20/14.55) | 900346 | 760235 | 10728 | 100.05 | Dengue-2 |
| MCL-21-H-11449 | Positive (21.75/21.10) | 4005842 | 89462 | 10733 | 100.09 | Dengue-2 |
| MCL-21-H-11634 | Positive (28.5 / 29.3) | 352446 | 1051 | 10561 | 98.49 | Dengue-2 |
| MCL-21-H-10601 | Positive (Ct Value 20.58/20.60) | 392566 | 169451 | 10712 | 99.90 | Dengue-2 |
| MCL-21-H-10906 | Positive (24.89/24.32) | 93152 | 1418 | 10267 | 95.75 | Dengue-2 |
| MCL-21-H-10974 | Positive (17.95/18.04) | 62552 | 6562 | 10548 | 98.37 | Dengue-2 |
| MCL-21-H-10977 | Positive (20.48/20.05) | 17372 | 4082 | 10608 | 98.93 | Dengue-2 |
| MCL-21-H-11204 | Positive (24.39/24.19) | 194536 | 3058 | 10568 | 98.55 | Dengue-2 |
| MCL-21-H-11369 | Positive (17.58/17.65) | 355350 | 266651 | 10741 | 100.17 | Dengue-2 |
| MCL-21-H-11400 | Positive (21.54/21.19) | 254718 | 147835 | 10719 | 99.96 | Dengue-2 |
| MCL-21-H-11408 | Positive (18.06/18.17) | 832912 | 713651 | 10733 | 100.09 | Dengue-2 |
| MCL-21-H-11428 | Positive (15.92/15.10) | 660242 | 79298 | 10466 | 97.60 | Dengue-2 |
| MCL-21-H-11443 | Positive (21.73/21.17) | 254732 | 16790 | 10236 | 95.46 | Dengue-2 |
| MCL-21-H-11653 | Positive ( 27.15/ 27) | 117608 | 494 | 9687 | 90.34 | Dengue-2 |
| MCL-21-H-12058 | Positive(19.48/19.33) | 912106 | 346880 | 10703 | 99.96 | Dengue-3 |
| MCL-21-H-11390 | Positive (22.59/22.47) | 937166 | 21054 | 10637 | 99.89 | Dengue-4 |
| MCL-21-H-11373 | Positive (19.27/19.20) | 207510 | 21103 | 10649 | 100.00 | Dengue-4 |
| MCL-21-H-11374 | Positive (23.28/23.22) | 146518 | 46716 | 10620 | 99.73 | Dengue-4 |
| MCL-21-H-11387 | Positive (23.98/23.87) | 148126 | 2195 | 10608 | 99.61 | Dengue-4 |
| MCL-21-H-11443 | Positive (21.73/21.17) | 254732 | 122698 | 10700 | 100.48 | Dengue-4 |
| MCL-21-H-12138 | Positive (22.45/22.13) | 217384 | 164332 | 10687 | 100.36 | Dengue-4 |
| MCL-21-H-11473 | Positive (29.95/29.72) | 259624 | 1436 | 10516 | 98.75 | Dengue-4 |
| MCL-21-H-11909 | Positive(28.97/29.56) | - | - | 421 | <98% | Zika |
| MCL-21-H-12032 | Positive(29.44/30.00) | - | - | 415 | <98% | Zika |
| MCL-21-H-12036 | Positive(31.39/34.34) | - | - | 415 | <98% | Zika |
| MCL-21-H-12013 | Positive(29.24/29.19) | - | - | 423 | <98% | Zika |
| MCL-21-H-11910 | Positive(32.53/32.07) | - | - | 412 | <98% | Zika |
| MCL-21-H-11934 | Positive(31.41/31.02) | - | - | 421 | <98% | Zika |
| MCL-21-H-11961 | Positive(32.61/32.17) | - | - | 419 | <98% | Zika |
| MCL-21-H-12034 | Positive(29.64/30.02) | - | - | 421 | <98% | Zika |
| MCL-21-H-12048 | Positive(31.62/31.27) | - | - | 416 | <98% | Zika |
| MCL-21-H-12053 | Positive(27.34/27.45) | - | - | 412 | <98% | Zika |
| MCL-21-H-11928 | Positive(30.55/30.77) | - | - | 422 | <98% | Zika |
| MCL-21-H-12047 | Positive(30.45/30.48) | - | - | 410 | <98% | Zika |
| MCL-21-H-12062 | Positive(31.88/31.22) | - | - | 408 | <98% | Zika |
| MCL-21-H-11913 | Positive(29.91/30.48) | - | - | 395 | <98% | Zika |
| MCL-21-H-12040 | Positive(28.87/29.67) | - | - | 402 | <98% | Zika |
| MCL-21-H-11969 | Positive(29.6/29.7) | - | - | 391 | <98% | Zika |
| MCL-21-H-12051 | Positive(28.24/29.16) | - | - | 390 | <98% | Zika |
| MCL-21-H-12057 | Positive(29.63/29.78) | - | - | 389 | <98% | Zika |
| MCL-21-H-11962 | Positive(32.25/32.00) | - | - | 369 | <98% | Zika |
| MCL-21-H-11919 | Positive(28.17/28.23) | - | - | 354 | <98% | Zika |
| MCL-21-H-11946 | Positive(32.13/31.89) | - | - | 360 | <98% | Zika |
| MCL-21-H-11947 | Positive(33.88/33.58) | - | - | 358 | <98% | Zika |
| MCL-21-H-12009 | Positive(30.47/30.09) | - | - | 357 | <98% | Zika |
| MCL-21-H-11915 | Positive(32.39/33.35) | - | - | 360 | <98% | Zika |
| MCL-21-H-11935 | Positive(32.53/33.17) | - | - | 349 | <98% | Zika |
| MCL-21-H-12029 | Positive(29.03/29.34) | - | - | 350 | <98% | Zika |
| MCL-21-H-11951 | Positive(31.92/31.79) | - | - | 358 | <98% | Zika |
| MCL-21-H-11949 | Positive(32.95/32.57) | - | - | 254 | <98% | Zika |
| MCL-21-H-12044 | Positive(31.75/34.42) | - | - | 278 | <98% | Zika |
| MCL-21-H-12021 | Positive(31.82/34.7) | - | - | 216 | <98% | Zika |
| MCL-21-H-12059 | Positive(28.56/28.24) | - | - | 425 | <98% | Zika |
| MCl-21-H-11953 | Positive (23.72/22.97) | 215066 | 18910 | 10673 | 98.88 | Zika |

**Supplementary Figure-1:** Changes in the amino acid sequences of Zika virus with respect to reference: NC 012532

**
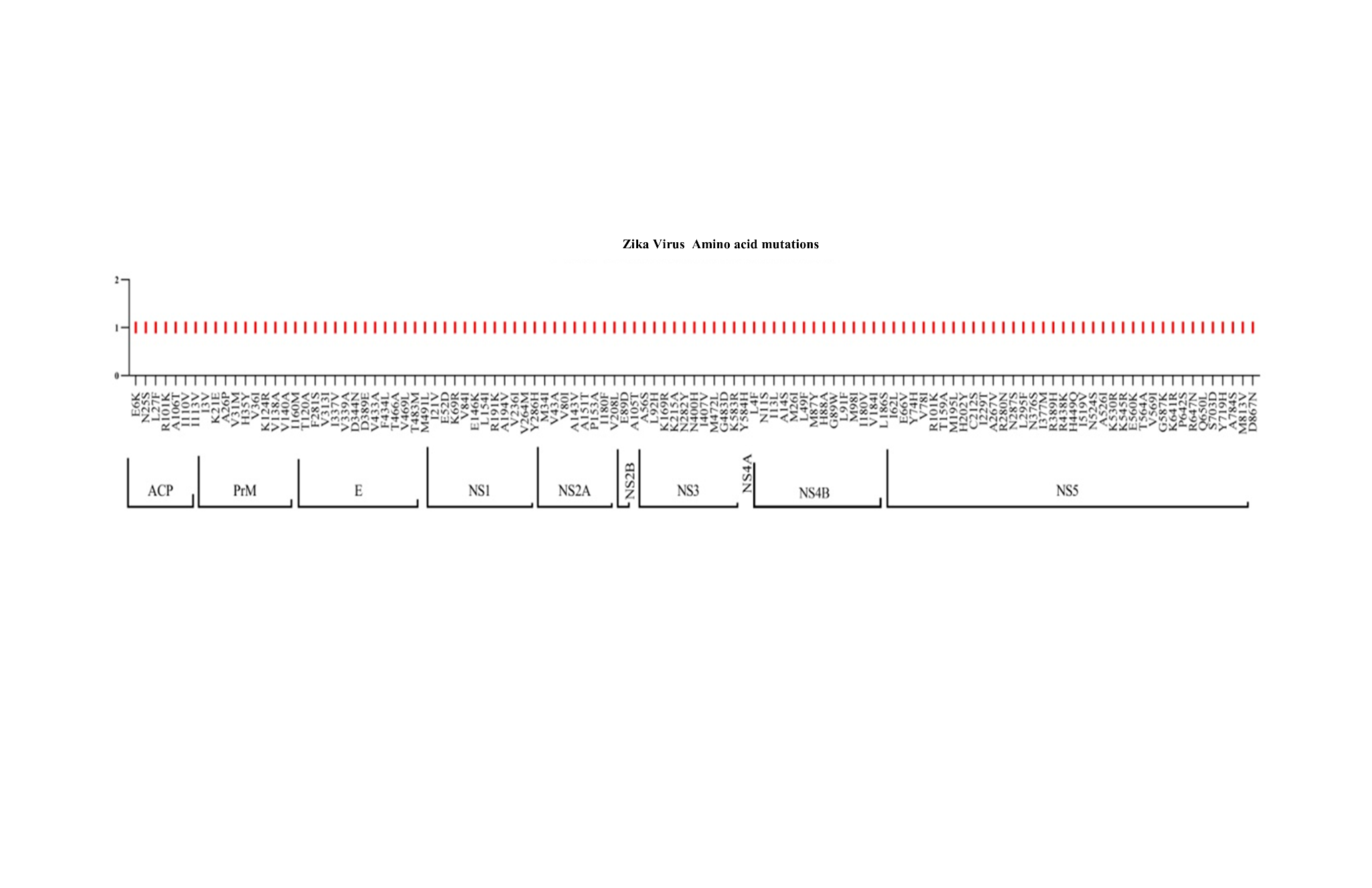
**

**Supplementary Figure-2:** Changes in the amino acid sequences of Dengue virus-1 in the NS5 gene retrieved in the study with respect to reference NC_001477


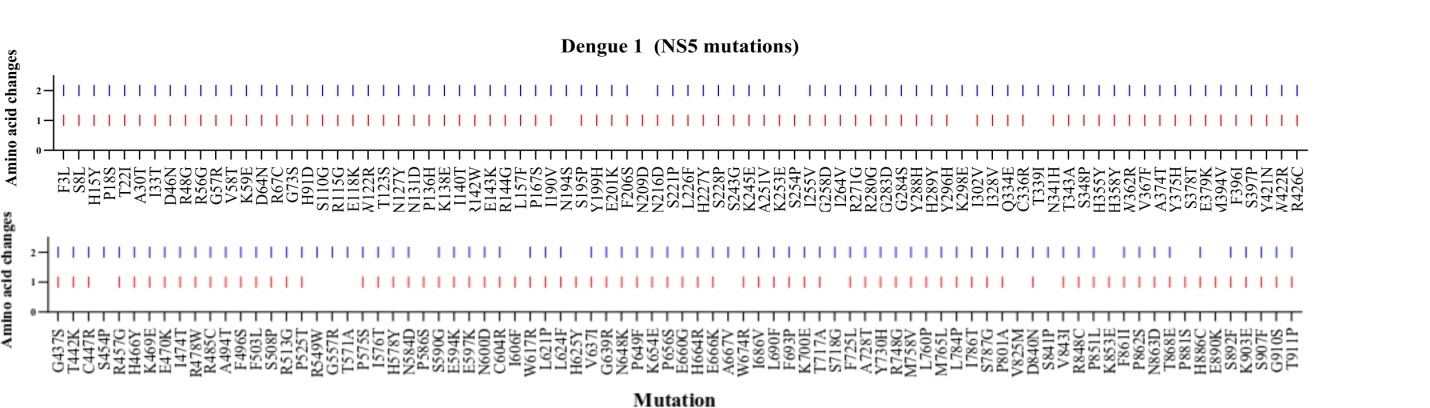


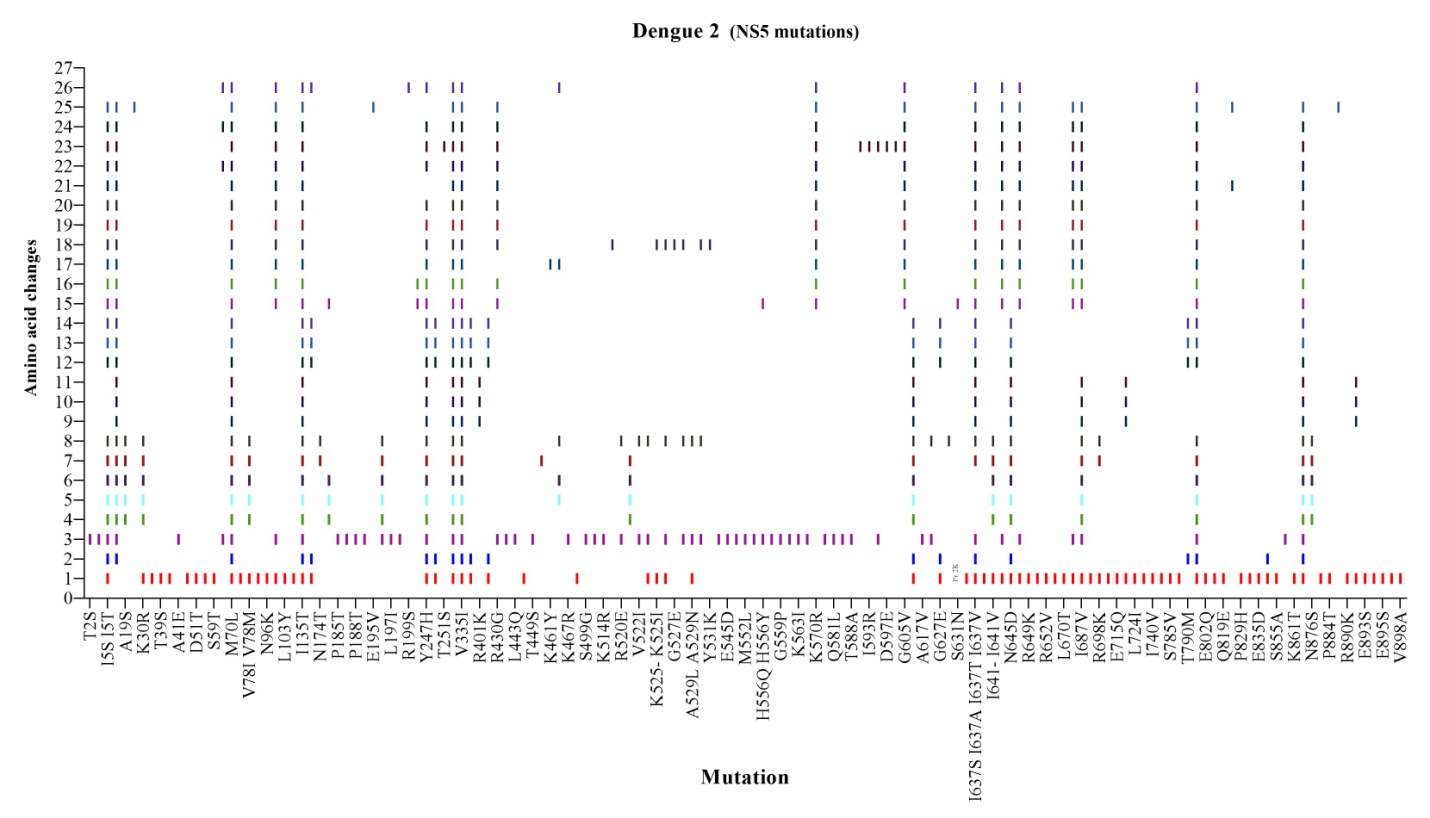
**Supplementary Figure-3:** Changes in the amino acid sequences of Dengue virus-2 in the NS5 gene retrieved in the study with respect to reference NC_001474

**Supplementary Figure-4:** Changes in the amino acid sequences of Dengue virus-3 in the NS5 gene retrieved in the study with respect to reference NC_001475


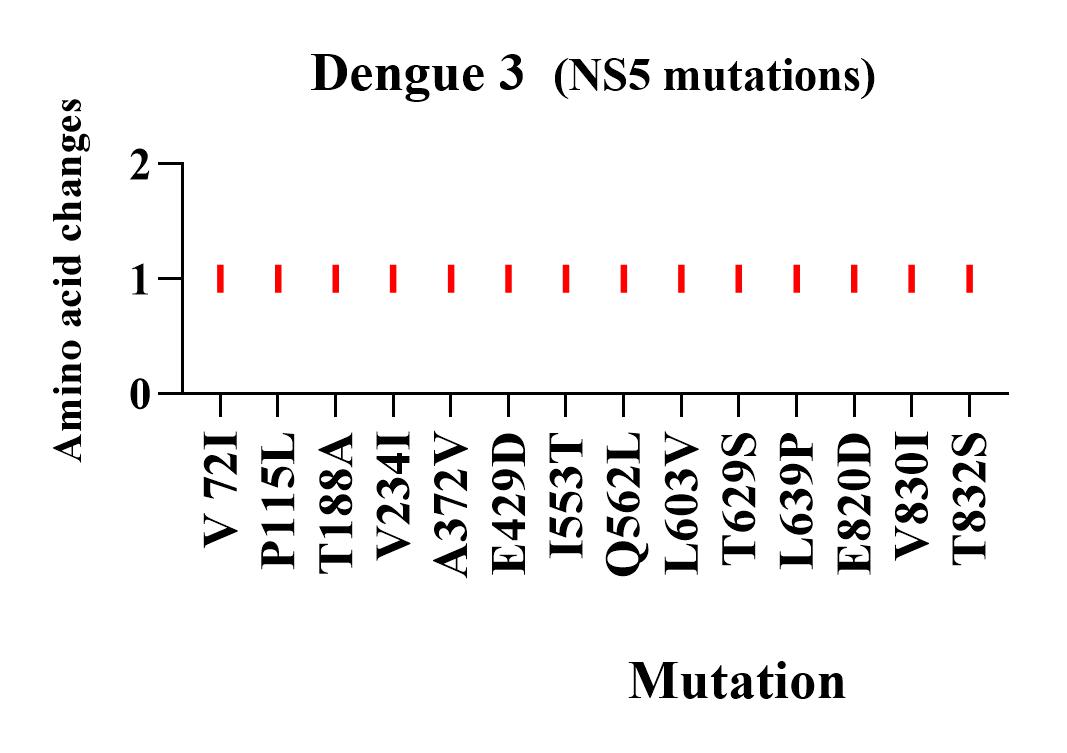


**Supplementary Figure-5:** Changes in the amino acid sequences of Dengue virus-4 in the NS5 gene retrieved in the study with respect to reference NC_002640.1


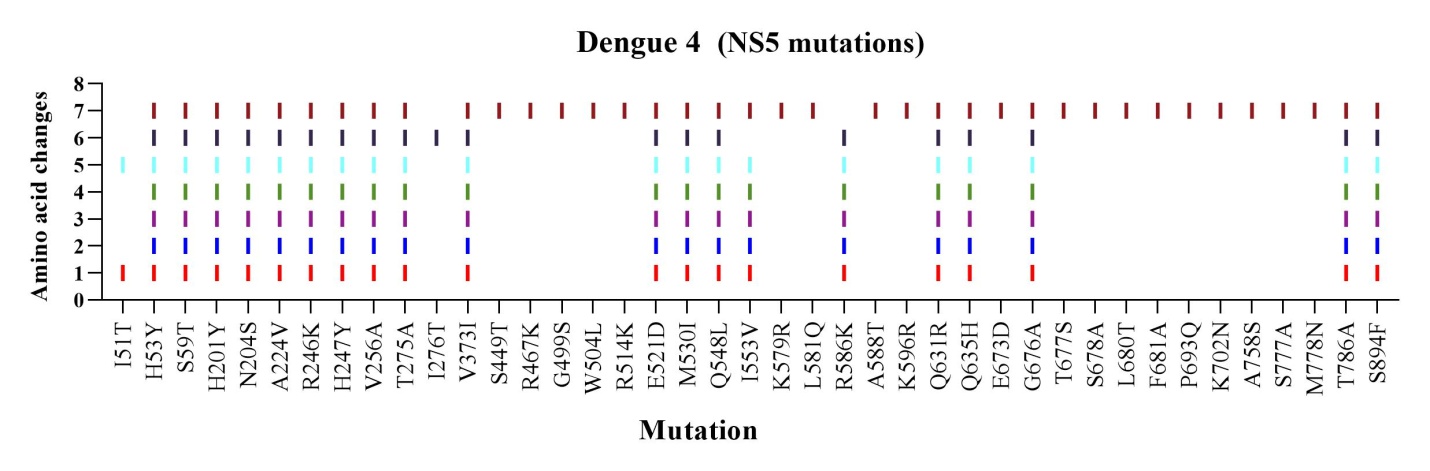

Supplement: Supplementary Figure 1 — Changes in the amino acid sequences of Zika virus with respect to reference: NC 012532. [file Table_2.DOC]
